# Self-reported confidence and perceived training needs of surgical interns at a regional hospital in Ghana: a questionnaire survey

**DOI:** 10.1186/s12909-020-02319-7

**Published:** 2020-10-27

**Authors:** Mee Joo Kang, Reuben Kwesi Sakyi Ngissah

**Affiliations:** 1 Center for Liver and Pancreatobiliary Cancer, National Cancer Center, 323 Ilsan-ro, Ilsandong-gu, Goyang-si, Gyeonggi-do 10408 Republic of Korea; 2 Department of Surgery, Greater Accra Regional Hospital, P.O. Box 473, Accra, Republic of Ghana

**Keywords:** Surgery, Surgical education, Curriculum, Confidence, Internship, Developing countries

## Abstract

**Background:**

Due to disparities in their regional distribution of the surgical specialists, those who have finished “housemanship,” which is the equivalent of an internship, are serving as main surgical care providers in rural areas in Ghana. However, the quantitative volume of postgraduate surgical training experience and the level of self-reported confidence after formal training have not been investigated in detail in sub-Saharan Africa.

**Methods:**

The quality-assessment data of the Department of surgery at a regional hospital in Ghana was obtained from the convenience samples of house officers (HOs) who had their surgical rotation before July 2019. A self-reported questionnaire with 5-point Likert-type scale and open-ended responses regarding the 35 topics listed as learning objectives by the Medical and Dental Council of Ghana were retrospectively reviewed to investigate the volume of surgical experience, self-reported confidence, and perceived training needs.

**Results:**

Among 52 respondents, the median self-reported number of patients experienced for each condition was less than 11 cases. More than 40% of HOs reported that they had never experienced cases of liver tumor (*n* = 21, 40.4%), portal hypertension (*n* = 23, 44.2%), or cancer chemotherapy/cancer therapy (*n* = 26, 50.0%). The median self-confidence score was 3.69 (interquartile range, 3.04 ~ 4.08). More than 50% of HOs scored ≤2 points on the self-confidence scale of gastric cancer (*n* = 28, 53.8%), colorectal cancer (*n* = 31, 59.6%), liver tumors (*n* = 32, 61.5%), and cancer chemotherapy/cancer therapy (*n* = 38, 73.1%). The top 3 reasons for not feeling confident were the limited number of patients (*n* = 42, 80.8%), resources and infrastructure (*n* = 21, 40.4%), and amount of supervision (*n* = 18, 34.6%). Eighteen HOs (34.6%) rated their confidence in their surgical skills as ≤2 points. Of all respondents, 76.9% (*n* = 40) were satisfied with their surgical rotation and 84.6% (*n* = 44) perceived the surgical rotation as relevant to their future work. Improved basic surgical skills training (*n* = 27, 51.9%) and improved supervision (*n* = 18, 34.6%) were suggested as a means to improve surgical rotation.

**Conclusions:**

Surgical rotation during housemanship (internship) should be improved in terms of cancer treatment, surgical skills, and supervision to improve the quality of training, which is closely related to the quality of surgical care in rural areas.

## Background

Inadequate infrastructure, supplies, and human resources for essential surgical care have been observed in Low-and-Middle-Income Countries (LMICs) [1]. Regarding human resources, a severe shortage of surgical specialist workforce has been widely emphasized [2]. In the World Health Organization (WHO) African region, the density of surgeons, anesthesiologists, and obstetricians was 1.0 per 100,000 population [3], which is far behind the target of 20 to 40 per 100,000 population [4]. Inequitable regional distribution of doctors within the country has also been pointed out [5]. For example, in Ghana, although the overall doctor-to-population ratio is decreasing, 58.3% of non-specialist doctors and 68.0% of specialists are working in two major regions of the country, where 35.4% of the overall population resides [6].

Apart from short-term surgical training of non-specialist physicians [7] or task-shifting to non-physician clinicians [8, 9], training more surgical specialists has been a main stem concerning the surgical workforce. To increase the specialist surgical workforce, postgraduate surgical training in Africa has been established by the West African College of Surgeons (WACS) [10], the Ghana College of Physicians and Surgeons (GCPS) [10], and the College of Surgeons of East, Central, and Southern Africa (COSECSA) [11]. In Ghana, although formal residency training is well-developed, a low number of physicians is entering surgical residency and only a few qualified surgeons are being trained every year. In addition, it is rare for surgical specialists to work in rural areas [12]. As a consequence, medical officers who have completed the period of housemanship are the main surgical care providers in Ghanaian district hospitals [13].

A recent systematic review reported that 19 of the 34 LMICs had an internship or housemanship program [14]. Although one must complete this mandatory period of training to earn a full medical license, the quality of training during this period has not been prioritized and there is little baseline data related to it. This period of training should be considered seriously because the rates of enrollment in residency programs are low in LMICs and the majority of doctors who work in rural areas are those who have just finished their housemanship or internship [12, 13]. However, it has been reported that medical officers experienced few supervised cases during their formal training, although they were responsible for performing major surgical procedures at district hospitals [12, 13].

Therefore, improving the quality of the surgical education of house officers (HOs) during housemanship would be a realistic and effective approach to improve the quality of surgical care, especially in rural areas. In this study, the authors report the HOs’ degree of experience and level of self-confidence in surgical conditions after surgical rotation to highlight the current status of HOs’ surgical competency and provide baseline data to improve the quality of surgical training.

## Methods

### Overview of housemanship in Ghana

In Ghana, the two-year housemanship period consists of four six-month rotations between internal medicine, obstetrics and gynecology, pediatrics, and surgery, and takes place shortly after medical school graduation [15]. During housemanship, two of the four rotations are done at teaching hospitals or their equivalents, including regional hospitals [15]. Emphasizing the importance of surgical rotation, the Medical and Dental Council (MDC) of Ghana has provided 35 conditions or procedures that can be managed by HOs “to prepare the house officer for safe and independent practice either in the community or health facility” [16].

### Study site

Located in the capital city Accra, the Greater Accra Regional Hospital (GARH) is one of the 10 regional hospitals in Ghana. The Department of surgery at GARH has 17 specialists (six general surgeons, four trauma and orthopedic surgeons, three urologists, two neurosurgeons, one pediatric surgeon, and one plastic and reconstructive surgeon). The annual volume of surgeries in the department is 1100 cases on average, 44% of cases being emergency operations. Among the specialties, the proportion of general surgery cases is the highest (43%), followed by trauma and orthopedic surgery (20%) and urology (13%). The department accommodates an average of 24 surgical HOs each term.

### Data collection

From July to August 2019, a quality assessment of the Department of Surgery was conducted with a convenience sample of HOs who had their surgical rotations before July 2019 at GARH. HOs voluntarily responded to the electronic evaluation form, which was anonymized and did not collect any personal identifiable information, such as name, age, gender, or personal contacts. The duration of the respondents’ surgical rotations, number of cases experienced, self-confidence regarding the 35 topics listed as learning objectives by the Medical and Dental Council of Ghana, and level of satisfaction with the surgical rotation were rated on a 5-point Likert-type scale. The details of the evaluation form are listed in Table 1. Concerning level of satisfaction and relevance, four or five points were considered satisfactory and relevant.

**Table 1:** Details of the evaluation form

Questions	Options and measures
**Demographics**	
Which year of housemanship are you in? ^a^	1st year
	2nd year
	Finished housemanship
How many months have you had surgical rotation? ^a^	Less than 2 months
	3 ~ 4 months
	5 ~ 6 months
In which hospital did you have your surgical rotation? ^a^	Open question
**MDC checklist (35 conditions or procedures)**	
How many cases have you experienced the following conditions/procedures? ^a^	Never
	1 ~ 5 cases
	6 ~ 10 cases
	11 ~ 20 cases
	More than 21 cases
Is there any condition/procedure you think not appropriate for housemanship? ^a^	Yes
	No
If yes, please name them and briefly explain the reason.	Open question
Is there any condition/procedure you want to add for the housemanship curriculum?	Open question
In what extent do you feel confident in managing the following conditions/procedures if you are on your own? ^a^	1 (not very confident) ~ 5 (very confident) in 5-point Likert-type scale
For the conditions/procedures that you don’t feel confident, please explain the reason. ^a^	Multiple selection for;
	Limited number of patients
	Limited supervision
	Limited resources and infrastructure
	Limited timeframe for surgical rotation
	Not interested in surgical conditions
	Other (free text)
**Level of satisfaction and suggestions**	
In overall, how satisfied were you with the surgical rotation? ^a^	1 (not very satisfied) ~ 5 (very satisfied) in 5-point Likert-type scale
How relevant and helpful do you think the surgical rotation was for your future work? ^a^	1 (not very relevant) ~ 5 (very relevant) in 5-point Likert-type scale
What were your key lessons learned during surgical rotation? ^a^	1 (not very confident) ~ 5 (very confident) in 5-point Likert-type scale for the following subdomains;
	Preoperative management
	Postoperative management
	Surgical skills
	Knowledge of basic principles
	Others
Please describe any suggestions to improve the quality of the surgical housemanship training	Open question

^a^Compulsory questions

### Data analysis

The quality assessment results were retrospectively reviewed to evaluate the surgical competency of the HOs during or after completing their surgical rotations and improve the quality of surgical training. Statistical analysis was conducted with STATA Version 15.1 (Stata Corp, Texas, USA). The aggregated scores for the number of cases experienced and self-confidence were derived from Likert-type scale scores in all 35 sub-domains. The summary measures were presented in median and interquartile ranges (IQR) because the aggregated scores did not follow normal distribution tested by Shapiro-Wilk W test and graphical inspection. The continuous variables were analyzed using the Wilcoxon rank-sum test and the nominal variables were analyzed using a chi-square test or Fisher’s exact test and Spearman correlation test. The two-sided *p* values < 0.05 were considered statistically significant. A thematic analysis was performed to analyze the results of the open-ended questions on the evaluation forms.

### Reliability and validity of the evaluation form

A reliability analysis was carried out on the questions, with 35 conditions and procedures rated in terms of the number of cases experienced and the respondents’ self-confidence regarding them. The internal consistency of the responses was evaluated with the Cronbach’s alpha value in each domain, which revealed that the questionnaire had an acceptable level of reliability (alpha value 0.969 [number of cases experienced] and 0.980 [self-confidence ratings]). As a consequence, all 35 topics were included for further analysis.

Construct validity of the level of satisfaction was demonstrated by positive correlation with the self-confidence score (Spearman’s correlation coefficient 0.392, *p =* 0.001). Although competence and confidence do not have a linear correlation [17], it is important for the HOs to achieve some degree of preparedness at least before they are posted to district or sub-district level hospitals as independent doctors. It is widely known that multiple factors affect self-efficacy beliefs in surgery [18], and case volume or length of training has a controversial correlation [17, 19]. However, the self-confidence of surgical trainees has been reported to have a positive correlation with level of satisfaction [20]. Therefore, the authors investigated the level of satisfaction as a measure of experience and self-confidence obtained during the surgical rotation.

## Results

### Characteristics

The overall response rate was 95.9% (71/74). Seven of the 71 respondents who had parts of their surgical rotation at other facilities (five at teaching hospitals, two at district-level hospitals) were excluded to investigate the authentic training status of a regional hospital. Fifty-two of the remaining 64 respondents who had completed 5 to 6 months of surgical rotation in GARH were included for final analysis. Each HO has a different order of rotations between the specialties. Seven (13.5%) were in their first year of housemanship, 11 (21.2%) were in their second year, and 34 (65.4%) had already finished the period of housemanship.

### Self-reported score for the number of cases experienced during surgical rotation

**Fig. 1:**
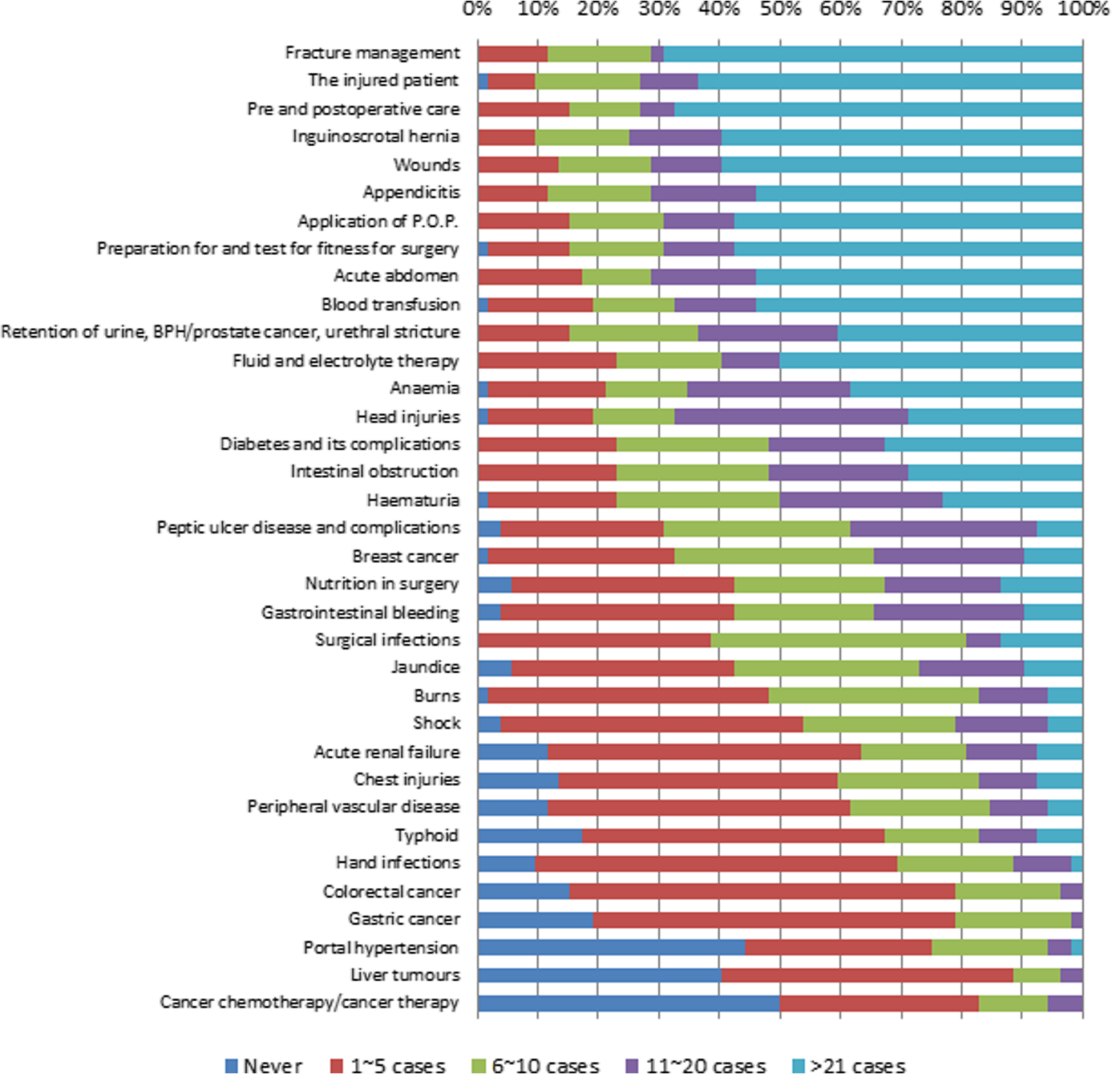
Self-reported score in number of the cases experienced

Although there is a risk of recall bias, the number of cases experienced was directly asked in the questionnaire because the majority of the HOs fill up their logbook at the end of the year with a higher risk of recall bias and inaccuracy. The median self-reported score for the number of cases experienced for each condition was 3.37 (IQR, 2.75 ~ 3.81). The median scores reported for each of the 35 conditions or procedures and the proportion of the HOs who had experienced five or fewer cases of them are shown in Table 2. The proportion of reported case scores for each topic is presented in Fig. 1. Eleven conditions or procedures were experienced in five or fewer cases in more than 50% of the HOs (shock, acute renal failure, chest injuries, peripheral vascular disease, typhoid, hand infections, colorectal cancer, gastric cancer, portal hypertension, liver tumors, and cancer chemotherapy/cancer therapy). More than 40% of the HOs reported that they had never experienced a case of liver tumor (*n* = 21, 40.4%), portal hypertension (*n* = 23, 44.2%), or cancer chemotherapy/cancer therapy (*n* = 26, 50.0%).

**Table 2:** Self-reported case scores and proportion of house officers who experienced ≤5 cases for the 35 items

Conditions or procedures	Case score (median, IQR)	Experienced ≤5 cases (*n* = 52)
Fracture management	5 (3, 5)	6 (11.5%)
The injured patient	5 (3, 5)	5 (9.6%)
Pre and postoperative care	5 (3, 5)	8 (15.4%)
Inguinoscrotal hernia	5 (3.5, 5)	5 (9.6%)
Wounds	5 (3, 5)	7 (13.5%)
Appendicitis	5 (3, 5)	6 (11.5%)
Application of P.O.P.	5 (3, 5)	8 (15.4%)
Preparation for and test for fitness for surgery	5 (3, 5)	8 (15.4%)
Acute abdomen	5 (3, 5)	9 (17.3%)
Blood transfusion	5 (3, 5)	10 (19.2%)
Retention of urine, including BPH/prostate cancer and urethral stricture	4 (3, 5)	8 (15.4%)
Fluid and electrolyte therapy	4.5 (3, 5)	12 (23.1%)
Anemia	4 (3, 5)	11 (21.2%)
Head injuries	4 (3, 5)	10 (19.2%)
Diabetes and its complications	4 (3, 5)	12 (23.1%)
Intestinal obstruction	4 (3, 5)	12 (23.1%)
Hematuria	3.5 (3, 4)	12 (23.1%)
Peptic ulcer disease and complications	3 (2, 4)	16 (30.8%)
Breast cancer	3 (2, 4)	17 (32.7%)
Nutrition in surgery	3 (2, 4)	22 (42.3%)
Gastrointestinal bleeding	3 (2, 4)	22 (42.3%)
Surgical infections	3 (2, 3)	20 (38.5%)
Jaundice	3 (2, 4)	22 (42.3%)
Burns	3 (2, 3)	25 (48.1%)
Shock	2 (2, 3)	28 (53.8%)
Acute renal failure	2 (2, 3)	33 (63.5%)
Chest injuries	2 (2, 3)	31 (59.6%)
Peripheral vascular disease	2 (2, 3)	32 (61.5%)
Typhoid	2 (2, 3)	35 (67.3%)
Hand infections	2 (2, 3)	36 (69.2%)
Colorectal cancer	2 (2, 2)	41 (78.8%)
Gastric cancer	2 (2, 2)	41 (78.8%)
Portal hypertension	2 (1, 2.5)	39 (75.0%)
Liver tumors	2 (1, 2)	46 (88.5%)
Cancer chemotherapy/cancer therapy	1.5 (1, 2)	43 (82.7%)

Liver tumors (*n* = 1) and portal hypertension (*n* = 1) were identified as items that could be removed from the checklist because of the limited number of cases. Surgical skills training with simulation or hands-on training in the operating theater (*n* = 1), splenectomy (*n* = 1), and cancer chemotherapy (*n* = 1) were identified as items that could be added to it.

### Self-reported confidence score

**Fig. 2:**
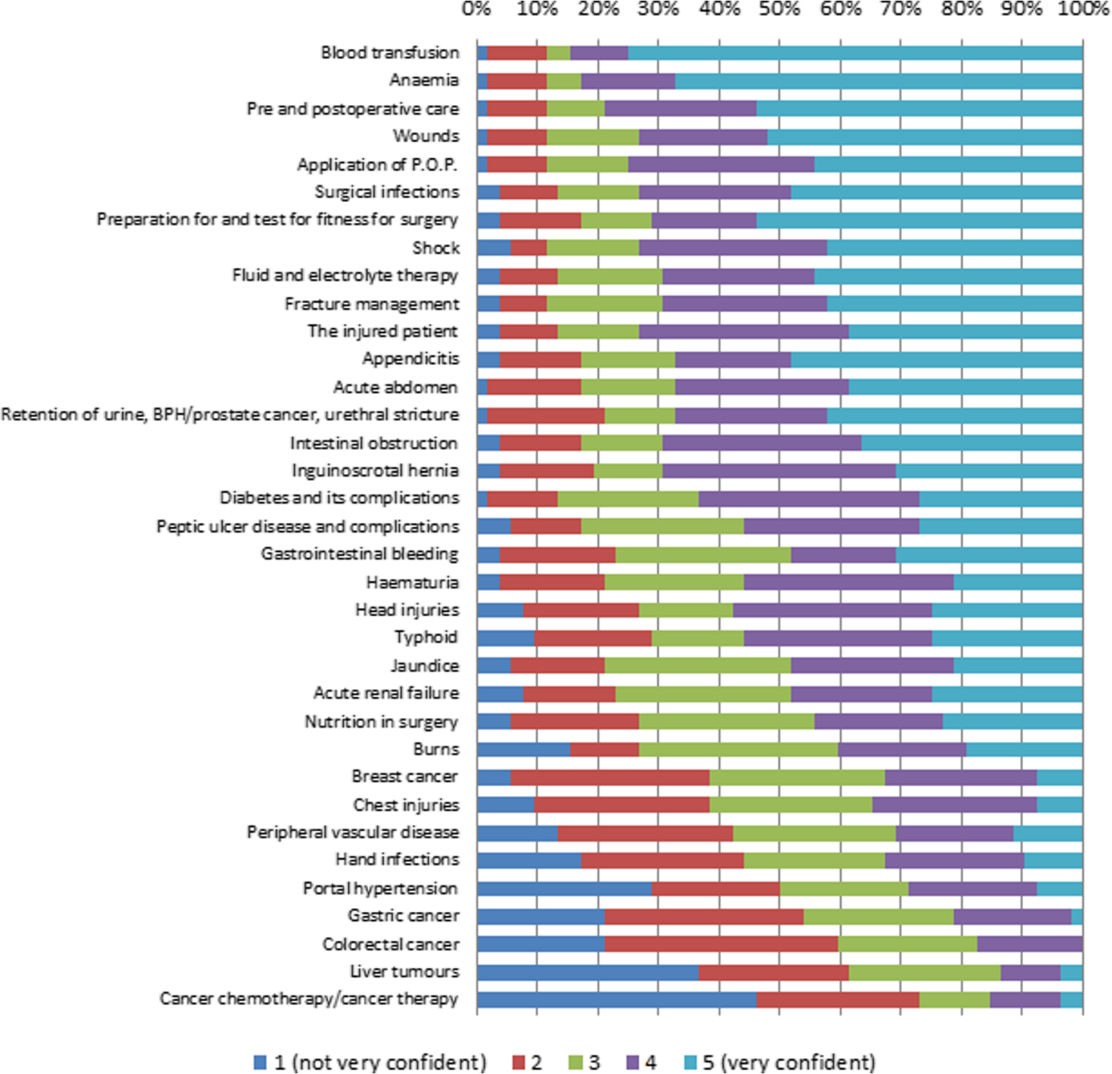
Self-reported confidence score

The median self-reported confidence score was 3.69 (IQR, 3.04 ~ 4.08). The median confidence scores reported for each of the 35 conditions or procedures and the proportion of the HOs who rated their self-confidence as 1 or 2 points are shown in Table 3. The proportions of reported confidence scores for each item are presented in Fig. 2. Four conditions scored 1 or 2 points on the self-confidence scale among more than 50% of HOs (gastric cancer, colorectal cancer, liver tumors, and cancer chemotherapy/cancer therapy). The self-reported confidence score was positively correlated with the self-reported score for the number of cases experienced (Spearman’s correlation coefficient 0.601, *p* < 0.001). Those who rated their confidence scores at 4 or 5 had higher self-reported scores for the number of cases experienced (median 3.66, IQR 3.47 ~ 4.09 [confidence score ≥ 4, *n* = 17] vs. median 3.09, IQR 2.66 ~ 3.60 [confidence score < 4, *n* = 35]; *p =* 0.011).

**Table 3:** Self-reported confidence scores and proportion of house officers who scored ≤2 points for the 35 items

Conditions or procedures	Confidence score (median, IQR)	Confidence score ≤ 2 (*n* = 52)
Blood transfusion	5 (4.5, 5)	6 (11.5%)
Anemia	5 (4, 5)	6 (11.5%)
Pre and postoperative care	5 (4, 5)	6 (11.5%)
Wounds	5 (3, 5)	6 (11.5%)
Application of P.O.P.	4 (3.5, 5)	6 (11.5%)
Surgical infections	4 (3, 5)	7 (13.5%)
Preparation for and test for fitness for surgery	5 (3, 5)	9 (17.3%)
Shock	4 (3, 5)	6 (11.5%)
Fluid and electrolyte therapy	4 (3, 5)	7 (13.5%)
Fracture management	4 (3, 5)	6 (11.5%)
The injured patient	4 (3, 5)	7 (13.5%)
Appendicitis	4 (3, 5)	9 (17.3%)
Acute abdomen	4 (3, 5)	9 (17.3%)
Retention of urine, including BPH/prostate cancer and urethral stricture	4 (3, 5)	11 (21.2%)
Intestinal obstruction	4 (3, 5)	9 (17.3%)
Inguinoscrotal hernia	4 (3, 5)	10 (19.2%)
Diabetes and its complications	4 (3, 5)	7 (13.5%)
Peptic ulcer disease and complications	4 (3, 5)	9 (17.3%)
Gastrointestinal bleeding	3 (3, 5)	12 (23.1%)
Hematuria	4 (3, 4)	11 (21.2%)
Head injuries	4 (2, 4.5)	14 (26.9%)
Typhoid	4 (2, 4.5)	15 (28.8%)
Jaundice	3 (3, 4)	11 (21.2%)
Acute renal failure	3 (3, 4.5)	12 (23.1%)
Nutrition in surgery	3 (2, 4)	14 (26.9%)
Burns	3 (2, 4)	14 (26.9%)
Breast cancer	3 (2, 4)	20 (38.5%)
Chest injuries	3 (2, 4)	20 (38.5%)
Peripheral vascular disease	3 (2, 4)	22 (42.3%)
Hand infections	3 (2, 4)	23 (44.2%)
Portal hypertension	2.5 (1, 4)	26 (50.0%)
Gastric cancer	2 (2, 3)	28 (53.8%)
Colorectal cancer	2 (2, 3)	31 (59.6%)
Liver tumors	2 (1, 3)	32 (61.5%)
Cancer chemotherapy/cancer therapy	2 (1, 3)	38 (73.1%)

Among the 11 items that the HOs had experienced five or fewer times during their surgical rotations, shock and typhoid had a relatively high proportion of HOs who rated their confidence scores at 4 or 5 (73.1 and 55.8%, respectively). Of the four items that elicited scores of 1 or 2 points in regard to self-confidence among more than 50% of HOs, more than 78% of HOs experienced five or fewer cases during surgical rotation (colorectal cancer *n* = 41, 78.8%; gastric cancer *n* = 41, 778.8%; cancer chemotherapy/cancer therapy *n* = 43, 82.7%; liver tumors *n* = 46, 88.5%). The reasons given for the lack of confidence towards these topics are listed in Table 4.

**Table 4:** Reasons for not feeling confident about the surgical conditions or procedures (multiple responses)

Theme	*N* = 52 (%)
Limited number of patients	42 (80.8%)
Limited resources and infrastructure	21 (40.4%)
Limited supervision	18 (34.6%)
Limited timeframe for surgical rotation	7 (13.5%)
Not interested in surgical conditions	2 (3.8%)

### Level of satisfaction and relevance to future work

Of all respondents, 76.9% (*n* = 40) were satisfied with their surgical rotation and 84.6% (*n* = 44) perceived the surgical rotation as relevant to their future work. The respondents’ level of satisfaction with the surgical rotation had a statistically significant correlation with the number of cases experienced, self-confidence, and perception of its relevance to their future work (Spearman’s correlation coefficient 0.286 [*p =* 0.040], 0.290 [*p =* 0.037], and 0.579 [*p* < 0.001], respectively).

### Key lessons learned during the surgical rotation

**Fig. 3:**
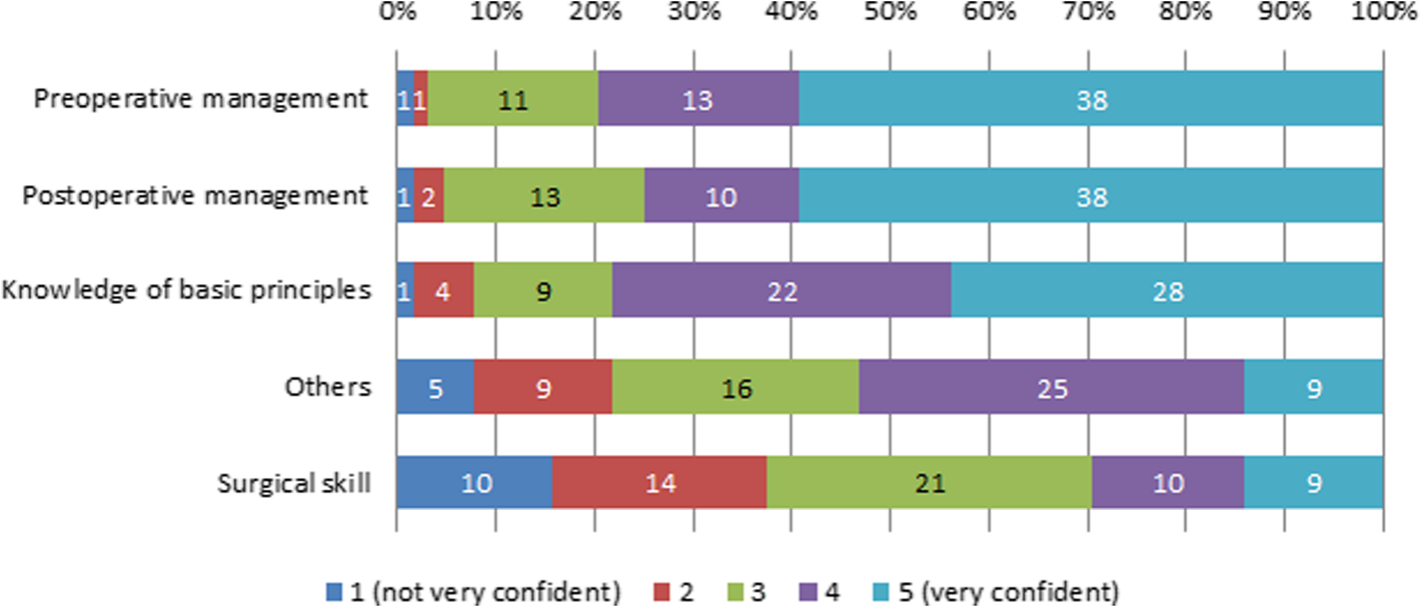
Key lessons learned during the surgical rotation

The areas in which respondents felt most confidence after their surgical rotations were preoperative management, followed by postoperative management, knowledge of basic principles, and surgical skills (Fig. 3). In regard to surgical skills, 34.6% (*n* = 18) of the respondents rated their confidence level as 1 or 2 points. Their level of confidence regarding surgical skills was not correlated with the mean self-reported number of cases experienced (*p =* 0.108). Those who had more confidence in their surgical skills perceived a higher level of satisfaction after the surgical rotation (Spearman’s correlation coefficient 0.375, *p =* 0.006).

### Suggestions from the HOs

Of the respondents, 36 (69.2%) HOs responded to the open-ended portion of the survey with their suggestions for improving the quality of the surgical rotation (Table 5). Twenty-seven (51.9%) suggested the introduction of basic surgical skills training, while 14 of them emphasized the need for hands-on training. A desire for more supervision in daily practice was expressed by 18 respondents (34.6%).

**Table 5:** Suggestions to improve quality of surgical rotation (multiple responses)

Theme	*N* = 36 (%)
Surgical skills, procedures, hands-on training at the operating theater	27 (75.0%)
Supervision and teaching	18 (50.0%)
Rotation in various subspecialties	1 (2.8%)

## Discussion

This study revealed that the HOs who had undergone training at the biggest regional hospital in Ghana experienced an average of six to 10 cases of each surgical condition or procedure during their 6-month rotations. The HOs had experienced more than six cases of benign conditions, including inguinoscrotal hernia, injury, appendicitis, acute abdomen, fracture management, intestinal obstruction, and peptic ulcer disease, with an average self-confidence score greater than 3.5. However, over 75% of the HOs had experienced five or fewer cases of cancer-related conditions (gastric cancer, colorectal cancer, liver tumors, and cancer chemotherapy/cancer therapy), with a self-confidence score lower than 2.5. As shown in the data, one of the main reasons for lack of confidence in treating the conditions was the limited number of patients with such conditions that the respondents had encountered. However, the learning objectives suggested by MDC are in accordance with cancer prevalence in the surgical field, which showed that breast cancer had the highest prevalence in Ghana, followed by liver, colorectal, and gastric cancer (71.3, 8.2, 7.9, and 3.8 cases per 100,000 population, respectively) [21]. The incidence of cancer in Africa is increasing rapidly because of changes in demographics and lifestyle as well as the urbanization of the population [22]. Due to the high prevalence of breast cancer, the HOs had experienced more than six cases of it, with a self-confidence score of 3. However, the regional hospital had inadequate case volumes for cancer treatment because the majority of cancer patients are referred to teaching hospitals. Therefore, training for cancer treatment should not be overlooked, and solutions such as case-scenario training or rotation in oncology units at teaching hospitals should be considered to improve the quality of surgical training [23]. In addition, the current state of training at teaching hospitals should be investigated to decide where and how long the rotation should take place.

The level of satisfaction with surgical rotation was higher among those who reported a higher level of perceived relevance of the surgical rotation to their future work. The HOs who perceived that the surgical rotation was more relevant to their future work may have participated more actively during the training, which may have resulted in a higher level of self-confidence and satisfaction. This observation highlights the importance of motivation, which is critical for successful learning [24]. However, external motivation should also be provided for effective training. A review of postgraduate medical education in sub-Saharan Africa suggested that unstructured programs, high workload and fatigue, and limited incentives for supervisors were challenges to conducting effective training [23]. In this study, 34.6% of the HOs rated their self-confidence in surgical skills as 1 or 2 points, and 51.9% of the respondents suggested a need for basic surgical skills training with hands-on experiences, reflecting their unmet needs in surgical skills training [23]. To improve preparedness among surgical interns, techniques like “shadowing” current interns in the United Kingdom and participating in specialty-focused surgical skills courses called “boot camps” in the United States have been utilized [25]. As a consequence, a weekly basic surgical skills training session that includes training in instrument handling, basic suturing techniques, and knot-tying commenced in 2019 at the Department of Surgery at GARH. However, the surgical procedures that the HOs must perform at district hospitals are beyond the scope of basic surgical skills [13]. Building on basic training, structured procedural skills training with exposure to real cases in the theater or emergency room is urgently needed.

On the other hand, 34.6% of respondents focused on the unsatisfactory supervision in daily practice. “Physically and mentally remote teachers” and “theoretical, inconsistent, and irrelevant teaching, such as grand rounds or didactic education sessions” have been reported as factors related to poor clinical practice [26]. However, unlike in developed countries, where the density of the specialist workforce is high, the massive workload caused by the paucity of specialists in LMICs makes it difficult for supervisors to maintain a strong passion for medical education. Supervisors’ competing responsibilities, including clinical practice and administrative work, and limited acknowledgment of such educational activities by colleagues or institutions, impede the prioritization of education [27]. Therefore, rather than relying on moral responsibility, systemic support, including peer recognition, career development, and various incentives, should be considered.

In addition, the MDC checklist included all of the “must do” procedures (laparotomy, treatment of open and closed fracture, and wound debridement) and parts of the “should do” (hernia repair, gastroscopy, cholecystectomy, intracranial hematoma evacuation, and mastectomy) and “can do” (chest injuries, retention of urine, and hematuria) procedures in the Lancet Commission on Global Surgery’s list in the field of general surgery [5]. However, some frequently performed “should do” procedures such as “superficial soft tissue tumor resection” or “thyroidectomy” were not listed in the MDC checklist, which requires reconsideration. Moreover, the majority of the current MDC checklist enlisted a broader concept of each condition, rather than specific surgical treatments or procedures, compared to the Lancet Commission on Global Surgery’s list. As a consequence, “should do” procedures were theoretically covered in the MDC checklist, but it is difficult to confirm that the HOs were trained to perform gastroscopy, cholecystectomy, intracranial hematoma evacuation, or mastectomy. Understanding the comprehensive concept of each disease entity cannot be overemphasized for entry-level doctors. However, refining the learning objectives into more specific and measurable items focused on surgery would help the HOs and supervisors to set an attainable goal during the surgical rotation, which should be different from internal medicine. Therefore, careful revision of the MDC checklist with specified minimum requirements for teaching cases may foster the development of a standardized curriculum with better involvement of HOs and supervisors dedicating their time for surgical skills training and education sessions [24].

Despite its valuable contributions, this study has several limitations. First, the anonymized format of the evaluation form made it impossible to conduct in-depth qualitative studies, such as face-to-face or focus group interviews, to enrich the analysis. In addition, studies of supervisors must be conducted to ascertain the main challenges they face during the supervision and education of HOs. Second, discrepancies between self-rated confidence and actual competency should be considered [28]. Objective assessment tools for procedural skills should be adopted to evaluate the surgical HOs’ competency in daily practice, and follow-up studies should incorporate this information. Lastly, the generalizability of this study’s results is limited because the study was based on a single center located in the capital city of a West African country. Based on this study, further investigation of HOs at various levels of institutions and in multiple regions may facilitate the development of a better curriculum and training policy from a national perspective.

Despite these limitations, this study explored the respondents’ degree of experience and self-confidence regarding surgical conditions after undergoing surgical rotation during housemanship, which is the only formal training that entry-level physicians receive before they are posted at district hospitals. Based on the data, further discussion, including approaches to increasing the motivation of HOs and improving the focus and quality of surgical rotation, is needed.

## Data Availability

The datasets used and analyzed during the current study are available from the corresponding author on reasonable request.

## Abbreviation

HO: House officer
